# Traditional Chinese medicine on treating dilated cardiomyopathy: A protocol for systematic review and meta analysis

**DOI:** 10.1097/MD.0000000000020777

**Published:** 2020-07-02

**Authors:** Yuqing Tan, Hengwen Chen, Jun Li, Qingjuan Wu, Xiaobo Wu, Wei Zhao

**Affiliations:** a Department of Cardiology, Guang’anmen Hospital, China Academy of Chinese Medical Sciences; b Beijing University of Chinese Medicine, Beijing, China.

**Keywords:** dilated cardiomyopathy, protocol, systematic review, traditional Chinese medicine

## Abstract

**Background:**

Dilated cardiomyopathy (DCM) is a type of complex cardiomyopathy characterized by enlargement and contractile dysfunction of the left ventricle, right ventricle, or double ventricle. Modern studies have shown that the pathogenesis of DCM is closely related to factors such as heredity, gene mutation, autoimmunity, and viral infection. The etiology is complex and the mortality rate is high. Many clinical trials have proved that traditional Chinese medicine has a great therapeutic effect on DCM. In this systematic review, we aim to evaluate the effectiveness and safety of traditional Chinese medicine for DCM.

**Methods:**

The databases of Pubmed, The Cochrane Library, Embase, China National Knowledge Infrastructure (CNKI), Wanfang Data Knowledge Service Platform (WANFANG Data), Weipu Information Chinese Periodical Service Platform (VIP), and China Biomedical Literature Service System (SinoMed) will be searched online to collect randomized controlled trials related to the treatment of DCM with Traditional Chinese medicine The time is limited from the construction of the library to December 2019. We will use the criteria provided by Cochrane 5.1.0 for quality assessment and risk assessment of the included studies, and use the Revman 5.3 and Stata 13.0 software so as to systematically review the effectiveness of Traditional Chinese medicine for DCM.

**Ethics and dissemination:**

This systematic review will evaluate the efficacy and safety of traditional Chinese medicine for DCM. Because all data used in this systematic review and meta-analysis have been published, this review does not require ethical approval. In addition, all data will be analyzed anonymously during the review process.

**Trial registration number:**

PROSPERO CRD42020163332.

## 1 Introduction

Dilated cardiomyopathy (DCM) is a kind of cardiomyopathy characterized by left ventricular or biventricular dilatation with systolic dysfunction,^[1]^ which could be accompanied by arrhythmia, thromboembolism, and sudden death.^[2]^ The pathogenesis of DCM is very complicated. DCM can be caused by a variety of causes, including genetics,^[3]^ gene mutation,^[1]^infection,^[4]^ substance abuse (e.g., alcohol and cocaine),^[5,6]^ non-infectious inflammation,^[7]^ and systemic endocrine or autoimmune diseases.^[8]^ Studies have used genetic screening suggest that up to 40% of DCM is genetically determined. Up to now, more than 50 genes encoding sarcomeric proteins, cytoskeleton, nuclear membrane, muscle membrane, ion channels, and intercellular connections have been involved in DCM.^[5]^ The most common is Titin-truncating variants (TTNtv), which has also been encountered in about 1% of the general population and they may remain silent.^[9]^

DCM develops in people of any age, gender, and race. In adults, the incidence of DCM is higher in men than in women. The annual incidence of children is 0.57 cases per 100,000 per year overall.^[4]^ It is believed that two thirds of children have idiopathic diseases. Among adults, the prevalence rate is one in 2500.^[10]^ Studies have shown that during the study period, the total risk of death or transplantation for idiopathic and familial DCM children is 30%, and 21% died in the first year after diagnosis.^[11]^ The 1-year mortality rate for adult patients with DCM is ∼25% to 30% and the 5-year survival rate is ∼50%.^[12]^ Due to the low age of onset of DCM, the complex pathogenesis and high mortality rate have brought a heavy burden on families and society. A statement from the European Society of Cardiology identifies two specific phenotypes of preclinical or early DCM: arrhythmic DCM and hypokinetic non-DCM.^[13]^ In addition to standard systolic heart failure treatment, treatment should be individualized and targeted at the underlying cause. In some cases, myocardial remodeling and cardiac dysfunction can be reversed by eliminating the cause and taking appropriate treatment measures.^[14]^ Congestive heart failure, circulatory failure, arrhythmia, and thromboembolic events are common in DCM. Treatment of chronic heart failure includes drugs that improve survival and reduce hospitalization, namely angiotensin-converting enzyme inhibitors and beta-blockers. Other interventions focus on symptoms and prevent complications. In addition, it includes stem cell therapy, mechanical circulation support, and heart transplantation.^[9]^

TCM is widely used clinically to treat DCM. TCM believes that DCM can belong to the category of “xinzhang.” Different doctors have different understandings of its etiology and pathogenesis. In short, TCM believes that the disease pathogenesis is asthenia in origin and excess in superficiality. The disease is closely related to the five internal organs, especially the heart. The interaction of various pathogenic factors such as qi stagnations, blood stasis, water dampness, phlegm-dampness, cold coagulation, etc leads to the disease.^[15]^ Studies have shown that Chinese medicine may improve cardiac function and reduce myocardial fibrosis by inhibiting the TGF-β1 signaling pathway. It also significantly reduces the volume fraction of myocardial collagen (CVF).^[16]^ Chinese patent medicines such as high-dose Tongxinluo can also effectively improve cardiac function in DCM rat models.^[17]^ Modern medicine is also studying the mechanism of TCM intervention in DCM from a pharmacological perspective.

Due to the limitation of clinical trial, most clinical trials are small samples and the evidence-based basis is relatively insufficient. Therefore, we expect a systematic review of randomized controlled trials of TCM for DCM, in order to determine the effectiveness of TCM combined with conventional western medicine in the treatment of DCM, and provide evidence-based evidence for clinical.

## 2 Methods

### 1.1 Study registration

This systematic review protocol has been registered on PROSPERO as CRD42020163332 (https://www.crd.york.ac.uk/prospero/display_record.php?ID=CRD42020163332) All of the data for the article have been published online, so this protocol does not require ethical approval. The protocol will be strictly enforced according the Guide to the contents of a Cochrane protocol and review (Part1.Chapter4 of Cochrane Handbook for systematic review of interventions Version 5.1.0).

### 1.2 Inclusion criteria for study selection

#### 1.2.1 Types of studies

We will search all the studies that traditional Chinese medicine is used as the main intervention for DCM. Non-randomized controlled trials (non-RCTs), quasi-randomized controlled trials (qRCTs), summaries of personal experience, case reports, and crossover studies will be excluded. The language of study is limited to Chinese and English.

#### 1.2.2 Types of participants

All the patients who have been diagnosed with DCM will be included. There are no restrictions on age, gender, regional, national, belief, ethnic, sources, and courses of disease.

#### 1.2.3 Types of interventions

There is no requirement for the intervention course, the specific contents of the control group and the experimental group are as follows.

##### 1.2.3.1 Control interventional

The control group takes conventional western medicine with diuretics, angiotensin-converting enzyme inhibitors, β-blockers, and other western medicines. The specific drugs, dosages, and methods are not limited. If the patients in the control group are treated with traditional Chinese medicine, the research will be excluded.

##### 1.2.3.2 Experimental interventional

The experimental group is treated with traditional Chinese medicine on the basis of conventional western medicine treatment in the control group. The use of traditional Chinese medicine is limited to prescription and Chinese patent medicines. Prescription drugs require a clear dose, but there are no restrictions on the composition, dosage form and dosage. For the dosage, such as decoctions, granules, pills, powders, etc. Other types of traditional Chinese medicine treatments such as traditional Chinese medicine injections, acupuncture, moxibustion, massage, cupping, and others will be excluded.

### 1.3 Outcome measures

#### 1.3.1 Primary outcomes

The primary outcomes are total efficiency rate (total efficiency rate = markedly effective rate + effective) and left ventricular ejection fraction (LVEF).

#### 1.3.2 Secondary outcomes

The secondary evaluation criteria are as follows: left ventricular end diastolic diameter (LVEDd); six-minute walk test (6MWT); heart rate (HR) and brain natriuretic peptide (BNP). More importantly, the incidence of adverse reactions and main adverse cardiovascular and cerebrovascular events (MACCE) in the trials will also be recorded.

### 1.4 Exclusion criteria for study selection

1. The studies without primary outcomes;
2. the studies that cannot be obtained in full text or no data can be extracted;
3. the studies with obvious errors in the data;
4. the one with the higher cases will be selected, while the studies have the same data source as the cases;
5. for duplicate publications or similar studies, just select one of them.

### 1.5 Searching strategy

#### 1.5.1 Electronic searches

The databases of PubMed, The Cochrane Library, Excerpt Medica Database (Embase), China National Knowledge Infrastructure (CNKI), Wanfang Data Knowledge Service Platform (WANFANG Data), Weipu Information Chinese Periodical Service Platform (VIP), and China Biomedical Literature Service System (SinoMed) will be searched online. The search time is set from the establishment of the search database to December 2019. According to the standards of the Cochrane Collaboration workbook of the International Evidence-Based Medicine Center, the search terms include “Chinese medicine,” “traditional Chinese medicine,” “proprietary Chinese medicine,” “Chinese herbal medicine,” “dilated cardiomyopathy,” and “xinzhang.” The complete PubMed search strategy is summarized in Table 1.

**Table 1:**
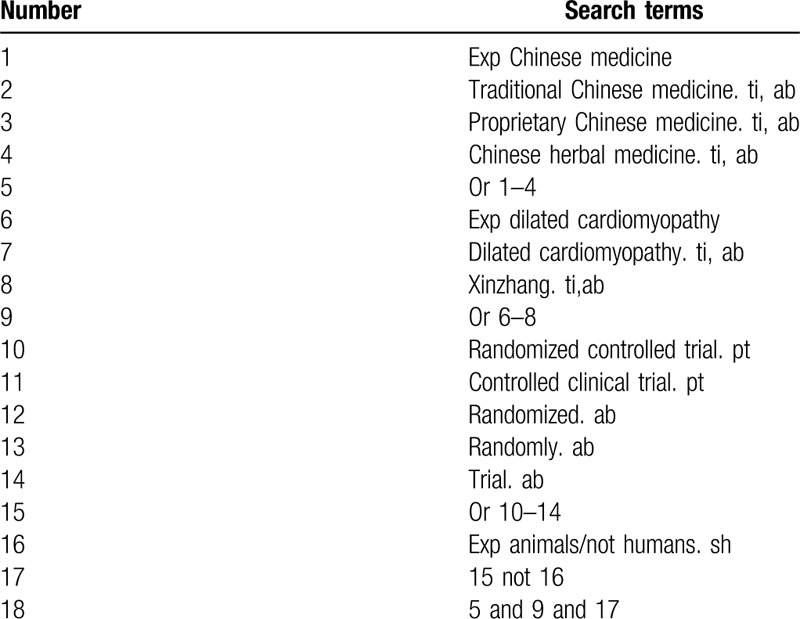
Search strategy used in PubMed database.

#### 1.5.2 Searching other resources

The manual is search mainly for ongoing experiments, and grey literature. We will look for abstracts and conference papers related to traditional Chinese medicine and DCM. Ongoing experiments which have been registered on World Health Organization International Clinical Trials Registry Platform (ICTRP) or Chinese Clinical Trial Registry. Try to contact the researchers to inquire about the progress of the trial and provide the latest test data. As for grey literature, we will retrieve on the following websites: GreyNet International, SIGLE (The System for Information on Grey Literature in Europe), Open Gery, Gery Literature Report. The secondary literature search will be performed on the included references to reduce the omission factor.

### 1.6 Data collection and analysis

#### 1.6.1 Study description

**Figure 1:**
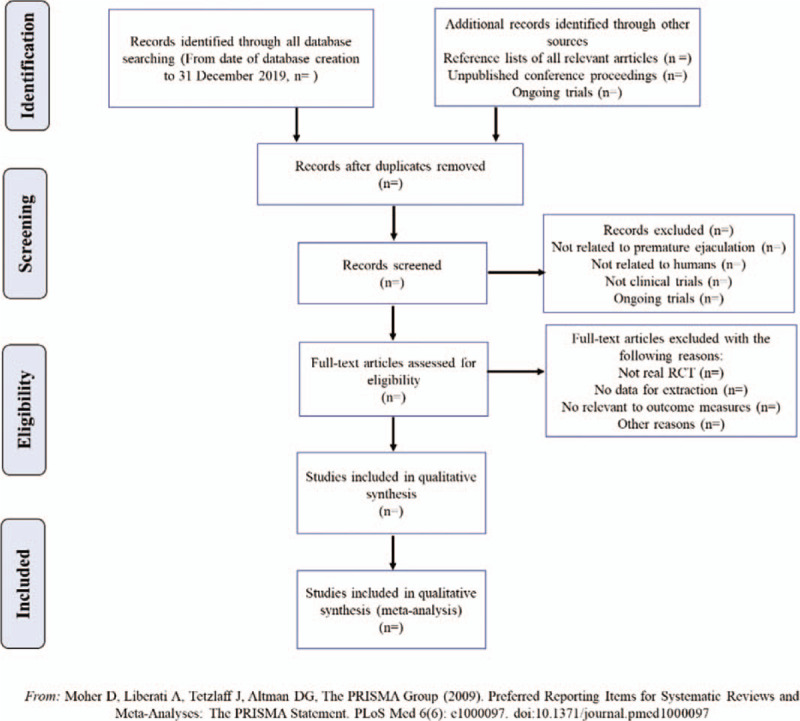
The PRISMA flow chart.

1. The initial screening: Retrieve several databases through the above search terms, and then import potentially relevant literature into Endnote X8. Researchers will use Endnote X8 to check duplicates and eliminate duplicate literature. Roughly browse documents to prevent careless omissions of the software.
2. The 2nd time of screening the literature: Two researchers will browse titles and abstracts based on inclusion and exclusion criteria, so as to exclude documents that are not related to the research, such as case reports, animal experiments, theoretical researches, studies on non-DCM. The details of selection process will be shown in the PRISMA flow chart in Figure 1.
3. The 3rd time of screening the literature: As for the remaining documents, download full texts. Two researchers will read them one by one to eliminate the substandard literature such as non-real RCT, lacking primary outcomes, fail to obtain the full text or valid data, literature whose intervention methods do not meet the criteria, etc. Also, the details of selection process will be shown in the PRISMA flow chart.

All the above processes are performed “back-to-back” by two researchers. If they encounter disagreement, the third researcher will resolve it through consultation or questioning. For documents where full text is not available, contact the corresponding author for full text. Read the full-text to determine whether to include it.

#### 1.6.2 Data extraction and management

First of all, a unified data extraction form will be developed. The data extraction of the literature is also independently completed by two researchers. This process can be completed with the help of excel software. The data extraction form is roughly as follows:

1. Basic information: article title, all authors, publication time, contact information. Article number uses first author's last name plus abbreviation of first name plus year of publication time, for example, related papers published by David Smith in September 2017 will be recorded as Smith D. 2017;
2. Research methods: diagnostic criteria, efficacy evaluation criteria, test type, sample size, generation of random number series, implementation of blind method, allocation concealment;
3. Participants: patient's age, gender, course of disease, location, number of cases in the control and experimental group;
4. Intervention method: the intervention method in the control group and experimental group of each literature, the dosage and frequency of medication, the intervention time, and the follow-up time;
5. Outcome measures: primary observation indicators, secondary observation indicators, improvement of indicators before and after treatment, detailed records of adverse reactions, major adverse cardiovascular and cerebrovascular events;
6. Source of funding and medical ethics review;
7. Others: number and reasons of dropouts or lost follow-up during the trial, etc.

#### 1.6.3 Risk of bias evaluation

As for the risk of bias in the literature, two researchers will independently use the tool for assessing risk of bias recommended by Cochrane Handbook for Systematic Reviews of Interventions 5.1.0 (Cochrane Handbook 5.1.0—Part 2: 8.5–8.7) to assess the quality of the included literature and risk of bias. Evaluation content includes: selection bias (random sequence generation, and allocation concealment), performance bias (blinding of participants and personnel), detection bias (blinding of outcome assessment), attrition bias (incomplete outcome data), reporting bias (selective outcome reporting), and other bias (other sources of bias). Evaluators judge the risk level by carefully reading the full text, which is divided into low risk, high risk and unknown risk. If the research reported in the literature is not detailed enough, the judgement is usually “unknown risk” of bias. For example, the study uses random number table for grouping, so the random sequence generation will be expressed as “low risk.” If there are any differences, we would consult the third reviewer for solution.

#### 1.6.4 Statistical analysis

The meta-analysis in this review will use RevMan 5.3 and Stata 13.0 software. For the outcome index of the two categorical variables, relative risk (Relativisk, RR) will be adopted, and for the outcome index of continuous variables, the mean difference (MD) or standardized mean difference (SMD) will be adopted will a confidence interval (CI) of 95%. Heterogeneity tests will be used for the included studies which will be tested by chi-square test. If *P* ≥ .10 and *I*^2^ ≤ 50%, there is no significant statistical heterogeneity or no statistical difference in heterogeneity, a fixed effect model will be adopted. If *P* < .10 and/or *I*^2^ > 50%, there is significant heterogeneity between studies, a random effect model will be adopted. Further analysis of the source of heterogeneity, if necessary, perform subgroup analysis. There are clinical and methodological differences in the experimental studies. Therefore, random effects models will be selected in this study. Finally, a funnel chart will be drawn to evaluate the publication bias of the literature.

#### 1.6.5 Publication bias

If a result of a meta-analysis contains more than 10 articles and above, we will use a funnel plot to test whether there is a publication bias. If the number of articles included in the study is <10, the publication bias is not significant.

#### 1.6.6 Quality of evidence

The quality of evidence will be assessed by Grades of Recommendations Assessment, Development and Evaluation (GRADE). The evaluation included: downgrade quality of evidence (risk of bias; inconsistency; indirectness; imprecision; publication bias) and upgrade quality of evidence (large effect; plausible confounding; dose–response gradient). The quality of evidence will be divided into four levels: high, moderate, low, and very low. Finally, refine the data and use software to edit, analyze, and draw summary of findings table.

## 3 Discussion

DCM is different from other diseases, and the age of onset tends to be younger, which has a great relationship with genetic factors. Chinese medicine believes that the pathogenesis of DCM is due to insufficiency of natural endowment, yang deficiency, and treatment based on the principle of supplementing qi and benefiting yang. TCM combined with conventional western medicine can effectively improve ejection fraction, reduce left ventricular end diastolic diameter, and improve exercise tolerance.^[18]^ Studies have shown that components of Radix Astragali can reduce myocardial inflammation and fibrosis, and protect heart function and morphological abnormalities.^[19]^ In recent years, due to the extensive development of clinical trials, the vigorous promotion of Chinese medicine has been applied to the clinic. TCM may be used as a complementary and alternative approach to primary and secondary prevention of cardiovascular disease.^[20]^ TCM has significant effects in treating heart diseases such as heart failure, arrhythmia, and coronary heart disease.

There is no uniform standard for the treatment of DCM, and clinicians have not reached a consensus. Due to the characteristics of DCM, the misdiagnosis rate is high. There are no large-scale epidemiological investigations for many reasons including incidence. The research on the mechanism of TCM to treat DCM has been progressing, but it is still not clear. We still need more efforts for diseases with unclear pathogenesis such as DCM. In recent years, there have been few systematic reviews and meta-analyses of TCM for the treatment of DCM, and there is no evidence-based medical evidence for the effectiveness and safety of TCM. Therefore, we hope to carry out this research, through a more comprehensive search of the database, expanding the sample size, and objectively evaluating the effectiveness and safety of traditional Chinese medicine in the intervention of DCM, in the hope of providing better diagnostic solutions for clinical doctors. However, due to the limitation of search setting conditions, such as language, search database, inclusion and exclusion criteria, etc, it will lead to some bias.

## Author contributions

**Data curation:** Yuqing Tan, Jun Li, Hengwen Chen.

**Formal analysis:** Yuqing Tan, Qingjuan Wu.

**Funding acquisition:** Jun Li, Hengwen Chen.

**Investigation:** Xiaobo Wu, Wei Zhao.

**Project administration:** Yuqing Tan, Jun Li.

**Resources:** Yuqing Tan, Jun Li, Hengwen Chen.

**Software:** Yuqing Tan, Qingjuan Wu, Jun Li, Hengwen Chen.

**Supervision:** Jun Li, Hengwen Chen.

**Validation:** Yuqing Tan, Jun Li, Hengwen Chen.

**Writing – original draft:** Yuqing Tan, Hengwen Chen.

**Writing – review & editing:** Jun Li, Hengwen Chen, Qingjuan Wu.
